# The genetic architecture of target‐site resistance to pyrethroid insecticides in the African malaria vectors *Anopheles gambiae* and *Anopheles coluzzii*

**DOI:** 10.1111/mec.15845

**Published:** 2021-03-08

**Authors:** Chris S. Clarkson, Alistair Miles, Nicholas J. Harding, Andrias O. O’Reilly, David Weetman, Dominic Kwiatkowski, Martin J. Donnelly, The Anopheles gambiae 1000 Genomes Consortium

**Affiliations:** ^1^ Wellcome Sanger Institute Cambridge UK; ^2^ Big Data Institute Li Ka Shing Centre for Health Information and Discovery University of Oxford Oxford UK; ^3^ Liverpool John Moores University Liverpool UK; ^4^ Liverpool School of Tropical Medicine Liverpool UK

**Keywords:** insecticides, malaria, mosquitoes, resistance, voltage‐gated sodium channel, whole genome sequencing

## Abstract

Resistance to pyrethroid insecticides is a major concern for malaria vector control. Pyrethroids target the voltage‐gated sodium channel (VGSC), an essential component of the mosquito nervous system. Substitutions in the amino acid sequence can induce a resistance phenotype. We use whole‐genome sequence data from phase 2 of the *Anopheles gambiae* 1000 Genomes Project (Ag1000G) to provide a comprehensive account of genetic variation in the *Vgsc* gene across 13 African countries. In addition to known resistance alleles, we describe 20 other non‐synonymous nucleotide substitutions at appreciable population frequency and map these variants onto a protein model to investigate the likelihood of pyrethroid resistance phenotypes. Thirteen of these novel alleles were found to occur almost exclusively on haplotypes carrying the known L995F *kdr* (knock‐down resistance) allele and may enhance or compensate for the L995F resistance genotype. A novel mutation I1527T, adjacent to a predicted pyrethroid‐binding site, was found in tight linkage with V402L substitutions, similar to allele combinations associated with resistance in other insect species. We also analysed genetic backgrounds carrying resistance alleles, to determine which alleles have experienced recent positive selection, and describe ten distinct haplotype groups carrying known *kdr* alleles. Five of these groups are observed in more than one country, in one case separated by over 3000 km, providing new information about the potential for the geographical spread of resistance. Our results demonstrate that the molecular basis of target‐site pyrethroid resistance in malaria vectors is more complex than previously appreciated, and provide a foundation for the development of new genetic tools for insecticide resistance management.

## 1 INTRODUCTION

Pyrethroid insecticides have been the cornerstone of malaria prevention in Africa for almost two decades (Bhatt et al., 2015). Pyrethroids are currently used in all insecticide‐treated bed‐nets (ITNs) and are used in indoor residual spraying (IRS) as well as in agriculture. Resistance to these insecticides is now widespread in malaria vector populations across Africa (Hemingway et al., 2016). The World Health Organization (WHO) has published plans for insecticide resistance management (IRM) that emphasize the need for improvements in both our knowledge of the molecular mechanisms of resistance and our ability to monitor them in natural populations (World Health Organization, 2012; World Health Organization et al., 2017).

The voltage‐gated sodium channel (VGSC) is the physiological target of pyrethroid insecticides and is integral to the insect nervous system. The sodium channel protein consists of four homologous domains (DI‐DIV) each of which comprises six transmembrane segments (S1–S6) connected by intracellular and extracellular loops (Dong et al., 2014). Pyrethroid molecules bind to this protein, stabilize the ion‐conducting active state and thus disrupt normal nervous system function, producing paralysis (‘knock‐down’) and death. However, amino acid substitutions at key positions within the protein alter the interaction with insecticide molecules, increasing the dose of insecticide required for knock‐down, known as knock‐down resistance or *kdr* (Davies et al., 2007a; Dong et al., 2014).

In the African malaria vectors *Anopheles gambiae* and *An. coluzzii*, three substitutions have been found to cause pyrethroid resistance. Two of these substitutions occur in codon 995^1^, with L995F prevalent in West and Central Africa (Martinez‐Torres et al., 1998; Silva et al., 2014), and L995S found in Central and East Africa (Ranson et al., 2000; Silva et al., 2014). A third substitution, N1570Y, has been found in West and Central Africa and shown to increase resistance in association with L995F (Jones et al., 2012). However, studies in other insect species have found a variety of other *Vgsc* substitutions inducing a resistance phenotype (Davies et al., 2007b; Dong et al., 2014; Rinkevich et al., 2013). To our knowledge, no studies in malaria vectors have analysed genetic variation across the full *Vgsc* coding sequence, and thus, the molecular basis of pyrethroid target‐site resistance has not been fully explored.

Basic information is also lacking about the spread of pyrethroid resistance in malaria vectors (World Health Organization, 2012). For example, it is not clear when, where or how many times pyrethroid target‐site resistance has emerged. Geographical paths of transmission, carrying resistance alleles between mosquito populations, are also not known. Previous studies have found evidence that L995F occurs on several different genetic backgrounds, suggesting multiple independent outbreaks of resistance driven by this allele (Etang et al., 2009; Lynd et al., 2010; Pinto et al., 2007; Santolamazza et al., 2015). However, these studies analysed only small gene regions in a limited number of mosquito populations and therefore had limited resolution to make inferences about relationships between haplotypes carrying this allele. It has also been shown that the L995F allele spread from *An. gambiae* to *An. coluzzii* in West Africa (Clarkson et al., 2014; Diabaté et al., 2004; Norris et al., 2015; Weill et al., 2000). However, both L995F and L995S now have wide geographical distributions (Silva et al., 2014), and to our knowledge, no attempts have been made to infer or track the geographical spread of either allele across Africa.

Here, we report an in‐depth analysis of genetic variation in the *Vgsc* gene, using whole‐genome Illumina sequence data from phase 2 of the *Anopheles gambiae* 1000 Genomes Project (Ag1000G) (The Anopheles gambiae 1000 Genomes Consortium, 2020). The Ag1000G phase 2 resource includes data on nucleotide variation in 1,142 wild‐caught mosquitoes sampled from 13 countries, with representation of West, Central, Southern and East Africa, and of both *An. gambiae* and *An. coluzzii*. We investigate variation across the complete gene coding sequence and report population genetic data for both known and novel non‐synonymous nucleotide substitutions. We then use haplotype data from the chromosomal region spanning the *Vgsc* gene to study the genetic backgrounds carrying resistance alleles, investigate the geographical spread of resistance between mosquito populations and provide evidence for recent positive selection. Finally, we explore ways in which variation data from Ag1000G can be used to design high‐throughput, low‐cost genetic assays for surveillance of pyrethroid resistance, with the capability to differentiate and track resistance outbreaks.

## 2 RESULTS

### 2.1 *Vgsc* non‐synonymous nucleotide variation

To identify variants with a potentially functional role in pyrethroid resistance, we extracted single nucleotide polymorphisms (SNPs) that alter the amino acid sequence of the VGSC protein from the Ag1000G phase 2 data resource (The Anopheles gambiae 1000 Genomes Consortium, 2020). We then computed their allele frequencies among 16 mosquito populations defined by species and country of origin. Alleles that confer resistance are expected to increase in frequency under selective pressure; therefore, we filtered the list of potentially functional variant alleles to retain only those at or above 5% frequency in one or more populations (Table 1). The resulting list comprises 23 variant alleles, including the known L995F, L995S and N1570Y resistance alleles, and a further 20 alleles which prior to Ag1000G had not previously been described in anopheline mosquitoes. We reported 12 of these novel alleles in our overall analysis of the 765 samples in the Ag1000G phase 1 data resource (The Anopheles gambiae 1000 Genomes Consortium, 2017), and we extend the analyses here to incorporate SNPs which alter codon 531, 697, 1507, 1603 and two tri‐allelic SNPs affecting codons 402 and 490.

**TABLE 1:** Non‐synonymous nucleotide variation in the voltage‐gated sodium channel gene

Variant				Population allele frequency (%)															
Position^a^	*Ag* ^b^	*Md* ^c^	Domain^d^	AO*Ac*	GH*Ac*	BF*Ac*	CI*Ac*	GN*Ac*	GW	GM	CM*Ag*	GH*Ag*	BF*Ag*	GN*Ag*	GA*Ag*	UG*Ag*	GQ*Ag*	FR*Ag*	KE
2,390,177 G > A	R254K	R261	IL45	0.0	0.009	0.0	0.0	0.0	0.0	0.0	0.313	0.0	0.0	0.0	0.203	0.0	0.0	0.0	0.0
2,391,228 G > C	V402L	V410	IS6	0.0	0.127	0.073	0.085	0.125	0.0	0.0	0.0	0.0	0.0	0.0	0.0	0.0	0.0	0.0	0.0
2,391,228 G > T	V402L	V410	IS6	0.0	0.045	0.06	0.0	0.0	0.0	0.0	0.0	0.0	0.0	0.0	0.0	0.0	0.0	0.0	0.0
2,399,997 G > C	D466H	‐	LI/II	0.0	0.0	0.0	0.0	0.0	0.0	0.0	0.069	0.0	0.0	0.0	0.0	0.0	0.0	0.0	0.0
2,400,071 G > A	M490I	M508	LI/II	0.0	0.0	0.0	0.0	0.0	0.0	0.031	0.0	0.0	0.0	0.0	0.0	0.0	0.0	0.0	0.188
2,400,071 G > T	M490I	M508	LI/II	0.0	0.0	0.0	0.0	0.0	0.0	0.0	0.003	0.0	0.0	0.0	0.0	0.0	0.0	0.0	0.0
2,402,466 G > T	G531V	G549	LI/II	0.0	0.0	0.0	0.0	0.0	0.0	0.0	0.0	0.0	0.0	0.0	0.007	0.0	0.056	0.0	0.0
2,407,967 A > C	Q697P	Q724	LI/II	0.0	0.0	0.0	0.0	0.0	0.0	0.0	0.0	0.0	0.0	0.0	0.0	0.0	0.056	0.0	0.0
2,416,980 C > T	T791M	T810	IIS1	0.0	0.009	0.02	0.0	0.0	0.0	0.0	0.0	0.292	0.147	0.112	0.0	0.0	0.0	0.0	0.0
2,422,651 T > C	L995S	L1014	IIS6	0.0	0.0	0.0	0.0	0.0	0.0	0.0	0.157	0.0	0.0	0.0	0.674	1.0	0.0	0.0	0.76
2,422,652 A > T	L995F	L1014	IIS6	0.84	0.818	0.853	0.915	0.875	0.0	0.0	0.525	1.0	1.0	1.0	0.326	0.0	0.0	0.0	0.0
2,429,556 G > A	V1507I	‐	IIIL56	0.0	0.0	0.0	0.0	0.125	0.0	0.0	0.0	0.0	0.0	0.0	0.0	0.0	0.0	0.0	0.0
2,429,617 T > C	I1527T	I1532	IIIS6	0.0	0.173	0.133	0.085	0.125	0.0	0.0	0.0	0.0	0.0	0.0	0.0	0.0	0.0	0.0	0.0
2,429,745 A > T	N1570Y	N1575	LIII/IV	0.0	0.0	0.267	0.0	0.0	0.0	0.0	0.057	0.167	0.207	0.088	0.0	0.0	0.0	0.0	0.0
2,429,897 A > G	E1597G	E1602	LIII/IV	0.0	0.0	0.0	0.0	0.0	0.0	0.0	0.0	0.0	0.065	0.062	0.0	0.0	0.0	0.0	0.0
2,429,915 A > C	K1603T	K1608	IVS1	0.0	0.055	0.047	0.0	0.0	0.0	0.0	0.0	0.0	0.0	0.0	0.0	0.0	0.0	0.0	0.0
2,430,424 G > T	A1746S	A1751	IVS5	0.0	0.0	0.0	0.0	0.0	0.0	0.0	0.0	0.292	0.141	0.1	0.0	0.0	0.0	0.0	0.0
2,430,817 G > A	V1853I	V1858	COOH	0.0	0.0	0.0	0.0	0.0	0.0	0.0	0.0	0.542	0.049	0.062	0.0	0.0	0.0	0.0	0.0
2,430,863 T > C	I1868T	I1873	COOH	0.0	0.0	0.0	0.0	0.0	0.0	0.0	0.0	0.0	0.261	0.2	0.0	0.0	0.0	0.0	0.0
2,430,880 C > T	P1874S	P1879	COOH	0.0	0.027	0.207	0.345	0.0	0.0	0.0	0.0	0.0	0.0	0.0	0.0	0.0	0.0	0.0	0.0
2,430,881 C > T	P1874L	P1879	COOH	0.0	0.0	0.073	0.007	0.25	0.0	0.0	0.0	0.0	0.234	0.475	0.0	0.0	0.0	0.0	0.0
2,431,061 C > T	A1934V	A1939	COOH	0.0	0.018	0.107	0.465	0.5	0.0	0.0	0.0	0.0	0.0	0.0	0.0	0.0	0.0	0.0	0.0
2,431,079 T > C	I1940T	I1945	COOH	0.0	0.118	0.04	0.0	0.0	0.0	0.0	0.067	0.0	0.0	0.0	0.0	0.0	0.0	0.0	0.0

Note

AO, Angola; GH, Ghana; BF, Burkina Faso; CI, Côte d'Ivoire; GN, Guinea; GW, Guinea‐Bissau; GM, Gambia; CM, Cameroon; GA, Gabon; UG, Uganda; GQ, Bioko; FR, Mayotte; KE, Kenya; *Ac*, *An. coluzzii*; *Ag*, *An. gambiae*. Species status of specimens from Guinea‐Bissau, Gambia and Kenya is uncertain (The Anopheles gambiae 1000 Genomes Consortium, 2020). All variants are at 5% frequency or above in one or more of the 16 Ag1000G phase 2 populations, with the exception of 2,400,071 G > T which is only found in the CM*Ag* population at 0.3% frequency but is included because another mutation is found at the same position (2,400,071 G > A) at >5% frequency and which causes the same amino acid substitution (M490I).

^a^ Position relative to the AgamP3 reference sequence, chromosome arm 2L.

^b^ Codon numbering according to *Anopheles gambiae* transcript AGAP004707‐RD in geneset AgamP4.12.

^c^ Codon numbering according to *Musca domestica* EMBL accession X96668 (Williamson et al., 1996).

^d^ Location of the variant within the protein structure. Transmembrane segments are named according to domain number (in Roman numerals) followed by ‘S’ then the number of the segment; for example, ‘IIS6’ means domain two, transmembrane segment six. Internal linkers between segments within the same domain are named according to domain (in Roman numerals) followed by ‘L’ then the numbers of the linked segments; for example, ‘IL45’ means domain one, linker between transmembrane segments four and five. Internal linkers between domains are named ‘L’ followed by the linked domains; for example, ‘LI/II’ means the linker between domains one and two. ‘COOH’ means the internal carboxyl tail.

**FIGURE 1:**
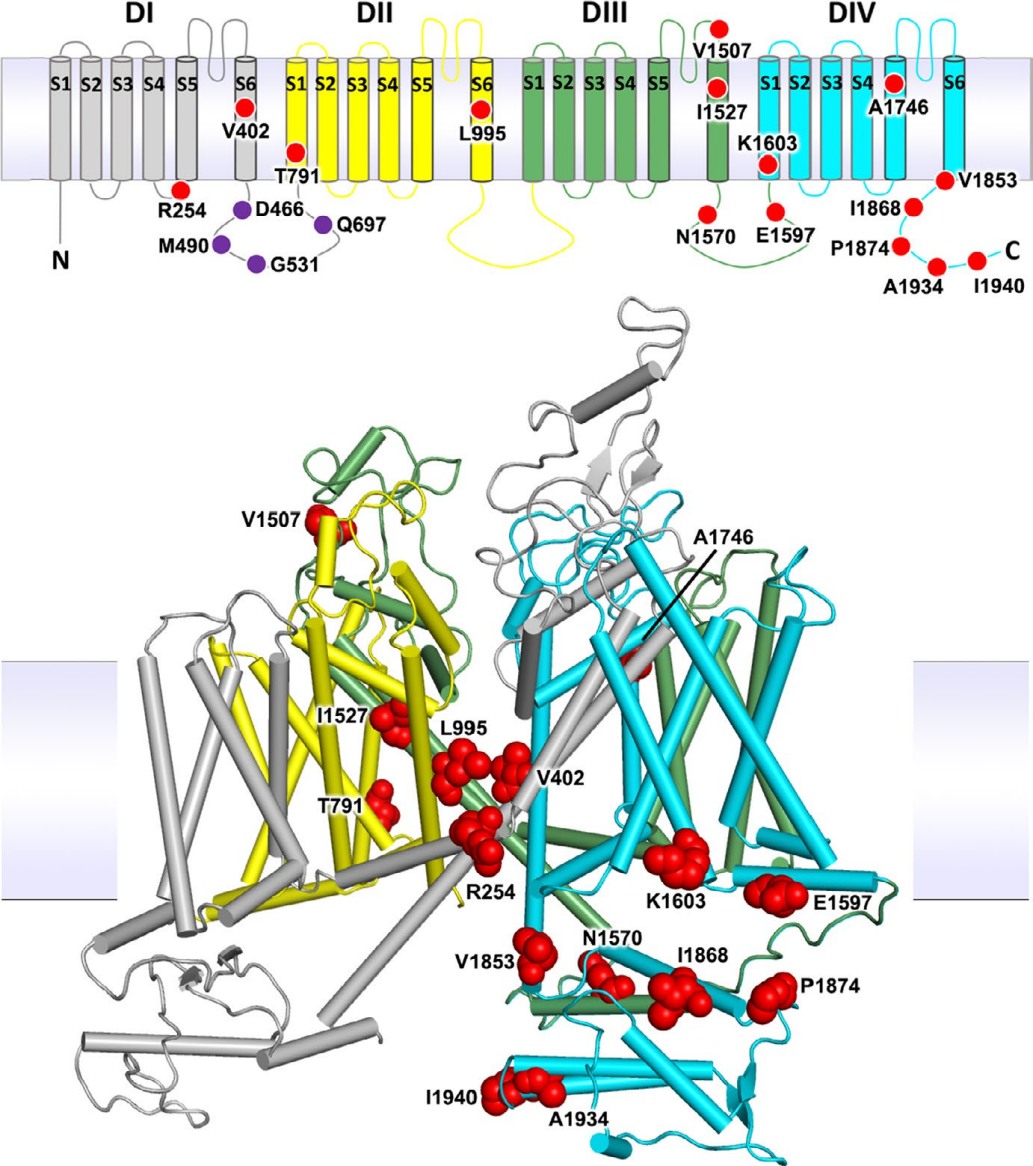
Voltage‐gated sodium channel protein structure and non‐synonymous variation. The *An. gambiae* voltage‐gated sodium channel (AGAP004707‐RD AgamP4.12) is shown as a transmembrane topology map (top) and as a homology model (bottom) in cartoon format coloured by domain. Variant positions are shown as red circles in the topology map and as red space‐fill in the 3D model. Purple circles in the map show amino acids absent from the model due to the lack of modelled structure in this region

The 23 non‐synonymous variants were located on a transmembrane topology map and on a 3‐dimensional homology model of the *Vgsc* protein. (Figure 1). The substitutions were found to be distributed throughout the channel, in all of the four internally homologous domains (DI‐DIV), in S1, S5 and S6 membrane‐spanning segments, in two of the intracellular loops connecting domains, and in the C‐terminal tail. The S5 and S6 segments that form the central ion‐conducting pore of the channel carry six of the eight segment substitutions, including V402 and L995 which have been shown to produce insecticide resistance phenotypes (Davies et al., 2007a; Dong et al., 2014; Martinez‐Torres et al., 1998; Ranson et al., 2000; Silva et al., 2014). Two substitutions are located on the DIII‐DIV linker including the resistance conferring N1570 (Jones et al., 2012). A further six substitutions are found concentrated in the protein's carboxyl tail (C‐terminus), including two alternative substitutions at the resistance‐associated P1874 residue (Sonoda et al., 2008). The DIII‐DIV linker and the C‐terminus segment interact in the closed‐state channel and substitutions are found throughout this intracellular subdomain. Finally, there are four novel substitutions located on the DI‐DII intracellular linker, but this region is missing from the model as it was not resolved in the cockroach NavPaS structure used as the model template (Shen et al., 2017).

The two known resistance alleles affecting codon 995 had the highest overall allele frequencies within the Ag1000G phase 2 cohort (Table 1). The L995F allele was at high frequency in populations of both species from West, Central and Southern Africa. The L995S allele was at high frequency among *An. gambiae* populations from Central and East Africa. Both of these alleles were present in *An. gambiae* populations sampled from Cameroon and Gabon. This included individuals with a heterozygous L995F/S genotype (50/297 individuals in Cameroon, 41/69 in Gabon). We calculated empirical *p*‐values for these heterozygous genotype counts using the Dirichlet distribution and 1,000,000 Monte Carlo simulations. In Cameroon, *p* = .410 of simulations found higher proportions of heterozygous genotypes; however, in Gabon this dropped to *p* = .005, suggesting there may be a fitness advantage for mosquitoes carrying both alleles in some circumstances.

**FIGURE 2:**
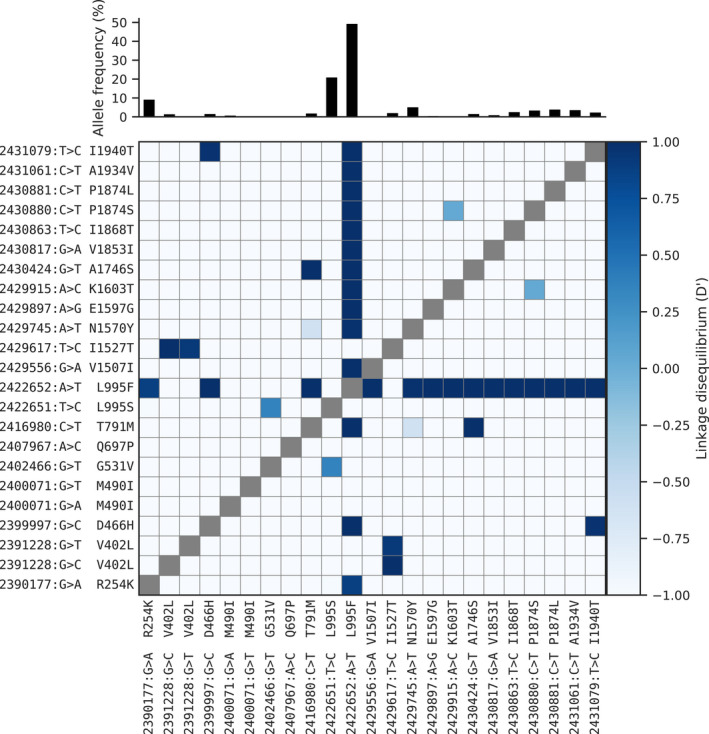
Linkage disequilibrium (*D*′) between non‐synonymous variants. A value of 1 indicates that two alleles are in perfect linkage, meaning that one of the alleles is only ever found in combination with the other. Conversely, a value of −1 indicates that two alleles are never found in combination with each other. The bar plot at the top shows the frequency of each allele within the Ag1000G phase 2 cohort. See Table 1 for population allele frequencies

The N1570Y allele was present in Guinea *An. gambiae*, Ghana *An. gambiae*, Burkina Faso (both species) and Cameroon *An. gambiae*. This allele has been shown to substantially increase pyrethroid resistance when it occurs in combination with L995F, both in association tests of phenotyped field samples (Jones et al., 2012) and functional tests using *Xenopus* oocytes (Wang et al., 2015). To study the patterns of association among non‐synonymous variants, we used haplotypes from the Ag1000G phase 2 resource to compute the normalized coefficient of linkage disequilibrium (*D*′) between all pairs of variant alleles (Figure 2). As expected, we found N1570Y in almost perfect linkage with L995F. Of the 20 novel non‐synonymous alleles, 13 also occurred almost exclusively in combination with L995F (Figure 2). These included two variants in codon 1874 (P1874S, P1874L), one of which (P1874S) has previously been associated with pyrethroid resistance in the crop pest moth *Plutella xylostella* (Sonoda et al., 2008).

The abundance of high‐frequency non‐synonymous variants occurring in combination with L995F is notable for two reasons. First, *Vgsc* is a highly conserved gene, expected to be under strong functional constraint and therefore purifying selection, so any non‐synonymous variants are expected to be rare (Davies et al., 2007b). Second, in contrast with L995F, we did not observe any high‐frequency non‐synonymous variants occurring in combination with L995S. This contrast was clear when data on all variants within the gene were considered: for haplotypes carrying the L995F allele, the ratio of non‐synonymous to synonymous nucleotide diversity *π_N_*/*π_S_* was 20.04 times higher than haplotypes carrying the wild‐type allele, but for those carrying L995S *π_N_*/*π_S_* was 0.5 times lower than haplotypes carrying the wild‐type allele. These results indicate that L995F has substantially altered the selective regime for other amino acid positions within the protein. Secondary substitutions have occurred and risen in frequency, suggesting that they are providing some further selective advantage in the presence of insecticide pressure.

A novel allele, I1527T, was present in *An. coluzzii* from Ghana, Burkina Faso, Cote d'Ivoire and Guinea. Codon 1527 occurs within trans‐membrane segment IIIS6, immediately adjacent to residues within a predicted binding site for pyrethroid molecules; thus, it is plausible that I1527T could alter pyrethroid binding (Dong et al., 2014; Du et al., 2013). We also found that the two variant alleles affecting codon 402, both of which induce a V402L substitution, were in strong linkage with I1527T (*D*′ ≥0.8; Figure 2), and almost all haplotypes carrying I1527T also carried a V402L substitution. Substitutions in codon 402 have been found in a number of other insect species and shown experimentally to confer pyrethroid resistance (Dong et al., 2014). The species and geographical distribution of the I1527T + V402L alleles suggest they arose in West African *An. coluzzii* and had not spread to other regions or to *An. gambiae* at the time of sampling. The I1527T allele was present at lower frequency than L995F in all of the West African *An. coluzzii* populations. L995F is known to have increased in frequency in West African *An. coluzzii* (Toé et al., 2014) and thus could be replacing I1527T + V402L in these populations. The four remaining novel alleles, Q697P, G531V and two separate nucleotide substitutions causing M490I, did not occur in combination with any known resistance allele and were almost exclusively private to a single population (Table 1).

### 2.2 Genetic backgrounds carrying resistance alleles

The Ag1000G data resource provides a rich source of information about the spread of insecticide resistance alleles in any given gene, because data are not only available for SNPs in protein coding regions, but also SNPs in introns, flanking intergenic regions and in neighbouring genes. These additional variants can be used to analyse the genetic backgrounds (haplotypes) on which resistance alleles are found. In our initial report of the Ag1000G phase 1 resource (The Anopheles gambiae 1000 Genomes Consortium, 2017), we used 1710 biallelic SNPs from within the 73.5 kbp *Vgsc* gene (1607 intronic, 103 exonic) to compute the number of SNP differences between all pairs of 1530 haplotypes derived from 765 wild‐caught mosquitoes. We then used pairwise genetic distances to perform hierarchical clustering and found that haplotypes carrying resistance alleles in codon 995 were grouped into 10 distinct clusters, each with near‐identical haplotypes. Five of these clusters contained haplotypes carrying the L995F allele (labelled F1‐F5), and a further five clusters contained haplotypes carrying L995S (labelled S1–S5).

**FIGURE 3:**
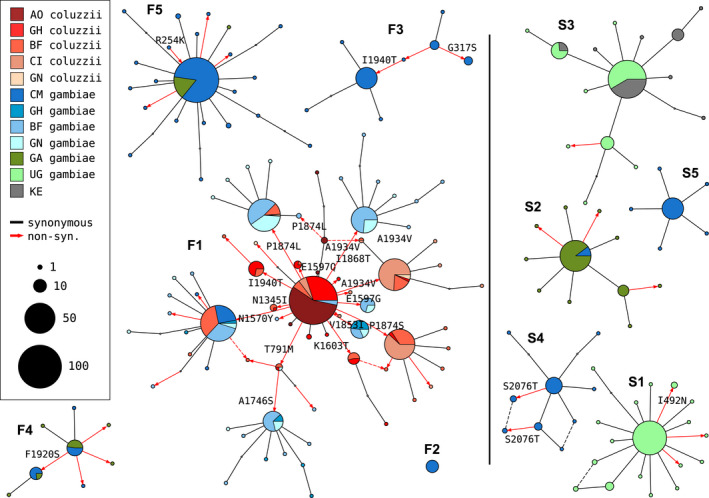
Haplotype networks. Median‐joining network for haplotypes carrying L995F (labelled F1‐F5) or L995S variants (S1–S5) with a maximum edge distance of two SNPs. Labelling of network components is via concordance with hierarchical clusters discovered in The Anopheles gambiae 1000 Genomes Consortium (2017). Node size is relative to the number of haplotypes contained, and node colour represents the proportion of haplotypes from mosquito populations/species—AO = Angola; GH = Ghana, BF = Burkina Faso; CI = Côte d'Ivoire; GN = Guinea; CM = Cameroon; GA = Gabon; UG = Uganda; KE = Kenya. Non‐synonymous edges are highlighted in red, and those leading to non‐singleton nodes are labelled with the codon change; arrow head indicates direction of change away from the reference allele. Network components with fewer than three haplotypes are not shown

To further investigate genetic backgrounds carrying resistance alleles, we used the Ag1000G phase 2 haplotype data from the *Vgsc* gene (2284 haplotypes from 1,142 mosquitoes (The Anopheles gambiae 1000 Genomes Consortium, 2020)), to construct median‐joining networks (Bandelt et al., 1999) (Figure 3). The network analysis improves on hierarchical clustering by allowing for the reconstruction and placement of intermediate haplotypes that may not be observed in the data. It also allows for non‐hierarchical relationships, which may arise if recombination events have occurred between haplotypes. We constructed the network up to a maximum edge distance of 2 SNP differences, to ensure that each connected component captures a group of closely related haplotypes. The resulting network contained 5 groups containing haplotypes carrying L995F, and a further 5 groups carrying L995S, in close correspondence with previous results from hierarchical clustering (96.8% overall concordance in assignment of haplotypes to groups).

The haplotype network brings into sharp relief the explosive radiation of amino acid substitutions secondary to the L995F allele (Figure 3). Within the F1 group, nodes carrying non‐synonymous variants radiate out from a central node carrying only L995F, suggesting that the central node represents the ancestral haplotype carrying just L995F which initially came under selection, and these secondary variants have arisen subsequently as new mutations. In F1 alone, 30 network edges (shown as red arrows—Figure 3) lead to non‐synonymous nodes. Many of the nodes carrying secondary variants are large, consistent with positive selection and a functional role for these secondary variants as modifiers of the L995F resistance phenotype. The F1 network also allows us to infer multiple introgression events between the two species. The central (putatively ancestral) node contains haplotypes from individuals of both species, as do nodes carrying the N1570Y, P1874L and T791M variants. This structure is consistent with an initial introgression of the ancestral F1 haplotype, followed later by introgressions of haplotypes carrying secondary mutations. The haplotype network also illustrates the contrasting levels of non‐synonymous variation between L995F and L995S. Within all of the L995S groups, only eight edges lead to non‐synonymous nodes and all these nodes are small (low‐frequency variants), thus may be neutral or mildly deleterious variants that are hitch‐hiking on selective sweeps for the L995S allele.

**FIGURE 4:**
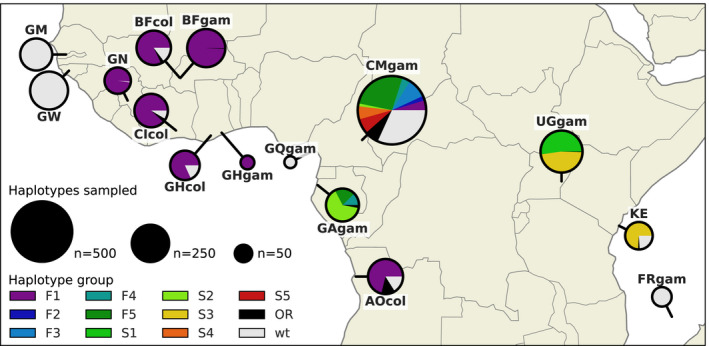
Map of haplotype frequencies. Each pie shows the frequency of different haplotype groups within one of the populations sampled. The size of the pie is proportional to the number of haplotypes sampled. The size of each wedge within the pie is proportional to the frequency of a haplotype group within the population. Haplotypes in groups F1‐F5 carry the L995F *kdr* allele. Haplotypes in groups S1–S5 carry the L995S *kdr* allele. Haplotypes in group other resistant (OR) carry either L995F or L995S but did not cluster within any of the haplotype groups. Wild‐type (*wt*) haplotypes do not carry any known resistance alleles

The F1 group contains haplotypes from mosquitoes of both species and from mosquitoes sampled in six different countries (Angola, Burkina Faso, Cameroon, Côte d'Ivoire, Ghana, Guinea) (Figure 4). The F4, F5 and S2 groups each contain haplotypes from both Cameroon and Gabon. The S3 group contains haplotypes from both Uganda and Kenya. The haplotypes within each of these five groups (F1, F4, F5, S2 and S3) were nearly identical across the entire span of the *Vgsc* gene (*π* < 4.5 × 10^−5^ bp^−1^). In contrast, diversity among wild‐type haplotypes was two orders of magnitude greater (Cameroon *An. gambiae* *π* = 1.4 × 10^−3^ bp^−1^; Guinea‐Bissau *π* = 5.7 × 10^−3^ bp^−1^). Thus, it is reasonable to assume that each of these five groups contains descendants of an ancestral haplotype that carried a resistance allele and has risen in frequency due to selection for insecticide resistance. Given this assumption, these groups each provide evidence for adaptive gene flow between mosquito populations separated by considerable geographical distances.

Populations carrying *kdr* alleles were collected between the years 2009 and 2012, with the exception of Gabon, which was collected in 2000. This temporal spread allows, albeit with low‐resolution, tracking of haplotypes through time. The spatially widespread F1 group contains haplotypes from samples collected between 2009 and 2012 (The Anopheles gambiae 1000 Genomes Consortium, 2020). We still do not know how fast insecticide resistance alleles can travel between these countries, but the large geographic spread suggests the F1 haplotype group originated some considerable time before the earliest collection in 2009. Haplotype groups F4, F5 and S2 all carry haplotypes from samples collected in Cameroon (2009) and Gabon (2000). These observations demonstrate that, even in mosquito populations with high levels of genetic diversity and large effective population size (The Anopheles gambiae 1000 Genomes Consortium, 2017), nucleotide sequences carrying alleles under strong selection can persist unchanged for almost a decade.

A limitation of both the hierarchical clustering and network analyses is that they rely on genetic distances within a fixed genomic window from the start to the end of the *Vgsc* gene. *Anopheles* mosquitoes undergo homologous recombination during meiosis in both males and females, and any recombination events that occurred within this genomic window could affect the way that haplotypes are grouped together in clusters or network components. In particular, recombination events could occur during the geographical spread of a resistance allele, altering the genetic background upstream and/or downstream of the allele itself. An analysis based on a fixed genomic window might then fail to infer gene flow between two mosquito populations, because haplotypes with and without a recombination event could be grouped separately, despite the fact that they share a recent common ancestor. To investigate the possibility that recombination events may have affected our grouping of haplotypes carrying resistance alleles, we performed a moving window analysis of haplotype homozygosity, spanning *Vgsc* and up to a megabase upstream and downstream of the gene (Figures S1 and S2). This analysis supported a refinement of our initial grouping of haplotypes carrying resistance alleles. All haplotypes within groups S4 and S5 were effectively identical on both the upstream and downstream flanks of the gene, but there was a region of divergence within the *Vgsc* gene itself that separated them in the fixed window analyses (Figure S3). The 13.8 kbp region of divergence occurred upstream of codon 995 and contained 6 SNPs that were fixed differences between S4 and S5. A possible explanation for this short region of divergence is that a gene conversion event has occurred within the gene, bringing a segment from a different genetic background onto the original genetic background on which the L995S resistance mutation occurred.

### 2.3 Positive selection for resistance alleles

**FIGURE 5:**
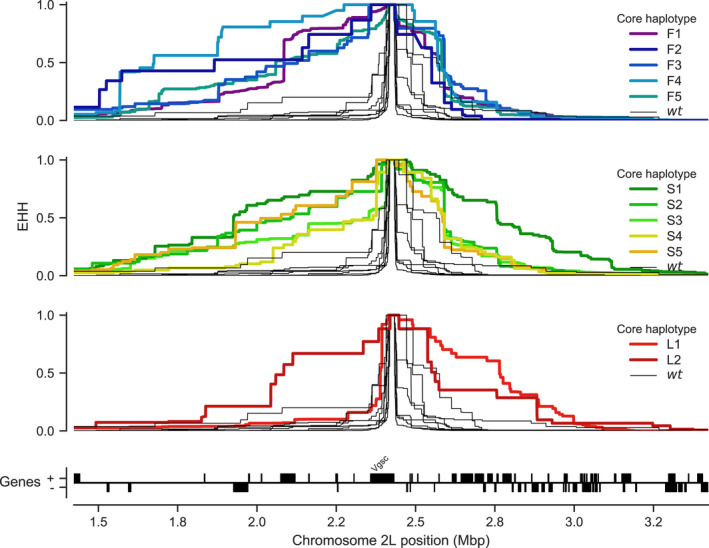
Evidence for positive selection on haplotypes carrying known or putative resistance alleles. Each panel plots the decay of extended haplotype homozygosity (EHH) for a set of core haplotypes centred on *Vgsc* codon 995. Core haplotypes F1‐F5 carry the L995F allele; S1–S5 carry the L995S allele; L1 carries the I1527T allele; L2 carries the M490I allele. Wild‐type (*wt*) haplotypes do not carry known or putative resistance alleles. A slower decay of EHH relative to wild‐type haplotypes implies positive selection (each panel plots the same collection of wild‐type haplotypes)

To investigate evidence for positive selection on non‐synonymous alleles, we performed an analysis of extended haplotype homozygosity (EHH) (Sabeti et al., 2002). Haplotypes under recent positive selection will have increased rapidly in frequency, thus have had less time to be broken down by recombination, and should on average have longer regions of haplotype homozygosity relative to wild‐type haplotypes. We defined a core region spanning *Vgsc* codon 995 and an additional 6 kbp of flanking sequence, which was the minimum required to differentiate the haplotype groups identified via clustering and network analyses. Within this core region, we found 18 distinct haplotypes at a frequency above 1% within the cohort. These included core haplotypes corresponding to each of the 10 haplotype groups carrying L995F or L995S alleles identified above, as well as a core haplotype carrying I1527T which we labelled L1 (due to it carrying the wild‐type leucine codon at position 995). We also found a core haplotype corresponding to a group of haplotypes from Kenya carrying an M490I allele, which we labelled as L2. All other core haplotypes we labelled as wild‐type (*wt*). We then computed EHH decay for each core haplotype up to a megabase upstream and downstream of the core locus (Figure 5).

As expected, haplotypes carrying the L995F and L995S resistance alleles all experience a slower decay of EHH relative to wild‐type haplotypes, supporting positive selection. Previous studies have found evidence for different rates of EHH decay between L995F and L995S haplotypes, suggesting differences in the timing and/or strength of selection (Lynd et al., 2010). However, we found no systematic difference in the length of shared haplotypes when comparing F1‐F5 (carrying L995F) against S1–S5 (carrying L995S) (Figure S3). There were, however, some differences between core haplotypes carrying the same allele. For example, shared haplotypes were significantly longer for S1 (median 1.006cM, 95% CI [0.986–1.040]) versus other core haplotypes carrying L995S (e.g. S2 median 0.593cM, 95% CI [0.589–0.623]; Figure S3). Longer shared haplotypes indicate a more recent common ancestor, and thus, some of these core haplotypes may have experienced more recent and/or more intense selection than others.

As sample collections took place over 12 years (2000–2012), it might be expected that core haplotypes appearing earlier in our sampling would have smaller shared haplotypes due to increased opportunity for recombination and mutation. However, no correlation was found between the year a core haplotype was first detected and the median length (*r*(8) = 0.03, *p* = 0.93, Figure S3).

The L1 haplotype carrying I1527T + V402L exhibited a slow decay of EHH on the downstream flank of the gene, similar to haplotypes carrying L995F and L995S, indicating that this combination of alleles has experienced positive selection. EHH decay on the upstream gene flank was faster, being similar to wild‐type haplotypes; however, there were two separate nucleotide substitutions encoding V402L within this group of haplotypes, and a faster EHH decay on this flank is consistent with recombination events bringing V402L alleles from different genetic backgrounds together with an ancestral haplotype carrying I1527T. The L2 haplotype carrying M490I exhibited EHH decay on both flanks comparable to haplotypes carrying known resistance alleles. This could indicate evidence for selection on the M490I allele, but these haplotypes are derived from a Kenyan mosquito population where there is evidence for a severe recent bottleneck (The Anopheles gambiae 1000 Genomes Consortium, 2017), and there were not enough wild‐type haplotypes from Kenya with which to compare. Thus, this signal may also be due to the extreme demographic history of this population.

## 3 DISCUSSION

### 3.1 Cross‐resistance between pyrethroids and DDT

The VGSC protein is the physiological target of both pyrethroid insecticides and DDT (Davies et al., 2007a). The L995F and L995S alleles are known to increase resistance to both of these insecticide classes (Martinez‐Torres et al., 1998; Ranson et al., 2000). By 2012, over half of African households owned at least one pyrethroid impregnated ITN and nearly two thirds of IRS programmes were using pyrethroids (Hemingway et al., 2016). Pyrethroids were also introduced into agriculture in Africa prior to the scale‐up of public health vector control programmes and continue to be used on a variety of crops such as cotton (Reid & McKenzie, 2016). DDT was used in Africa for several pilot IRS projects carried out during the first global campaign to eradicate malaria, during the 1950 s and 1960 s (Davies et al., 2007b). DDT is still approved for IRS use by WHO and remains in use in some locations; however within the last two decades, pyrethroid use has been far more common and widespread. DDT was also used in agriculture from the 1940 s, and although agricultural usage has greatly diminished since the 1970 s, some usage remains (Abuelmaali et al., 2013). In this study, we reported evidence of positive selection on the L995F and L995S alleles, as well as the I1527T + V402L combination and possibly M490I. We also found 14 other non‐synonymous substitutions that have arisen in association with L995F and appear to be positively selected. Given that pyrethroids have dominated public health insecticide use for two decades, it is reasonable to assume that the selection pressure on these alleles is primarily due to pyrethroids rather than DDT. It has previously been suggested that L995S may have been initially selected by DDT usage (Lynd et al., 2010). However, we did not find any systematic difference in the extent of haplotype homozygosity between these two alleles, suggesting that both alleles have been under selection over a similar time frame. We did find some significant differences in haplotype homozygosity between different genetic backgrounds carrying resistance alleles, suggesting differences in the timing and/or strength of selection these may have experienced. However, there have been differences in the scale‐up of pyrethroid‐based interventions in different regions, and this could in turn generate heterogeneities in selection pressures. Nevertheless, it is possible that some if not all of the alleles we have reported provide some level of cross‐resistance to DDT as well as pyrethroids, and we cannot exclude the possibility that earlier DDT usage may have contributed at least in part to their selection. The differing of resistance profiles to the two types of pyrethroids (type I, e.g., permethrin; and type II, e.g., deltamethrin) (Hu et al., 2011) will also affect the selection landscape. Further sampling and analysis will be required to investigate the timing of different selection events and relate these to historical patterns of insecticide use in different regions.

### 3.2 Resistance phenotypes for novel non‐synonymous variants

The non‐synonymous variants are distributed throughout the channel protein but can be considered in terms of three clusters: (a) the transmembrane domain, (b) the DI‐I intracellular linker and (c) the DIII‐DIV/C‐terminal subdomain. The pyrethroid‐binding site is located in the transmembrane domain between the IIS4‐S5 linker and the IIS5, IIS6 and IIIS6 helices (O’Reilly et al., 2006). The I1527T substitution that we discovered in *An. coluzzii* mosquitoes from Burkina Faso occurs in segment IIIS6 and is immediately adjacent to two pyrethroid‐sensing residues in this binding site (Dong et al., 2014). It is thus plausible that pyrethroid binding could be altered by this substitution. The I1527T substitution (*M. domestica* codon 1532) has been found in *Aedes albopictus* (Xu et al., 2016), and substitutions in the nearby codon 1529 (*M. domestica* I1534T) have been reported in *Aedes albopictus* and in *Aedes aegypti* where it was found to be associated with pyrethroid resistance (Dong et al., 2014; Ishak et al., 2015; Li et al., 2018). We found the I1527T allele in tight linkage with two alleles causing a V402L substitution (*M. domestica* V410L). Substitutions in codon 402 have been found in multiple insect species and are by themselves sufficient to confer pyrethroid resistance (Dong et al., 2014). The fact that we find I1527T and V402L in such tight mutual association is intriguing because haplotypes carrying V402L alone should also have been positively selected and thus be present in one or more populations.

The V402 residue is located towards the middle of the IS6 helix. The L995F and L995S substitutions occur at a similar position on the IIS6 helix. It was proposed these S6 substitutions confer resistance by allosterically modifying formation of the pyrethroid‐binding site (O’Reilly et al., 2006). More recently, the L995 *kdr* residue was speculated to form part of a second pyrethroid‐binding site in the insect channel termed ‘PyR2’ (Du et al., 2013, 2015). A major functional effect of the L995F substitution is enhanced closed‐state inactivation (Vais et al., 2000). This contributes to *kdr* resistance by reducing the number of channels that undergo activation, which is the functional state that pyrethroids bind to with highest affinity (Vais et al., 2000). Fast inactivation involves movement of the DIV domain to form a receptor for the DIII‐DIV linker fast inactivation particle containing the ‘MFM’ sequence motif (equivalent to the ‘IFM’ motif in mammals) (Capes et al., 2013; Dong et al., 2014). Recent eukaryotic sodium channel structures reveal that the DIII‐DIV linker is in complex with the C‐terminal segment in the closed‐state conformation but the DIII‐DIV linker appears to dissociate and bind in close proximity in the DIV S6 helix upon transition to the inactivated state (Shen et al., 2017; Yan et al. 2017). It seems that binding of the DIII‐DIV linker pushes the DIV S6 helix forward to occlude the pore and produce the inactivated state (Yan et al. 2017). We suggest that substitutions located on the DIII‐DIV linker and C‐terminal tail may perturb the conformation of this subdomain when it assembles in the closed‐state channel and may subsequently affect capture or release of the DIII‐DIV linker from this complex. The expected functional outcome would be altered channel inactivation, although whether inactivation is enhanced or diminished and if this compensates for a deleterious effect of L995F on channel function awaits elucidation. The N1570Y substitution on the DIII‐DIV linker has been functionally characterised but inactivation kinetics in the mutant channel were found unaltered (Wang et al., 2015). Pyrethroid sensitivity was also unaffected by N1570Y although resistance was greatly enhanced in the N1570Y + L995F double mutant (Wang et al., 2015).

The final cluster of novel variants is located on the DI‐DII intracellular linker. This segment includes the novel M490I substitution that was found on the Kenyan L2 haplotypic background potentially under selection. M490I did not occur in association with L995F or any other non‐synonymous substitutions. Although we were unable to model this region, we speculate that the DI‐DII linker passes under the DII S4–S5 linker and these regions may interact, as was found in a bacterial sodium channel structure (Sula et al., 2017). The structural effects of DI‐DII substitutions may be altered interactions with the DII S4–S5 linker, the movement of which is critical for formation of the pyrethroid‐binding site (O’Reilly et al., 2006; Usherwood et al., 2007). Overall, there are a number of potential mechanisms by which a pyrethroid resistance phenotype may arise and topology modelling reveals how many of the non‐synonymous variants we discover may be involved, though clearly much remains to be unravelled regarding the molecular biology of pyrethroid resistance in this channel.

### 3.3 Design of genetic assays for surveillance of pyrethroid resistance

Entomological surveillance teams in Africa regularly genotype mosquitoes for resistance alleles in *Vgsc* codon 995 and use those results as an indicator for the presence of pyrethroid resistance alongside results from insecticide resistance bioassays. They typically do not, however, sequence the gene or genotype any other polymorphisms within the gene. Thus, if there are other polymorphisms within the gene that cause or significantly enhance pyrethroid resistance, these will not be detected. Also, if a codon 995 resistance allele is observed, there is no way to know whether the allele is on a genetic background that has also been observed in other mosquito populations, and thus no way to investigate whether resistance alleles are emerging locally or being imported from elsewhere. Whole‐genome sequencing of individual mosquitoes clearly provides data of sufficient resolution to answer these questions and could be used to provide ongoing resistance surveillance. The cost of whole‐genome sequencing continues to fall, making it a practical tool for malaria vector surveillance. However, to achieve substantial spatial and temporal coverage of mosquito populations, it would also be necessary to develop targeted genetic assays for resistance outbreak surveillance. Technologies such as amplicon sequencing (Kilianski et al., 2015) are already being trialled on mosquitoes (Lucas et al., 2019), and these could scale to tens of thousands of samples at low cost and could be implemented using existing platforms in national molecular biology facilities.

**FIGURE 6:**
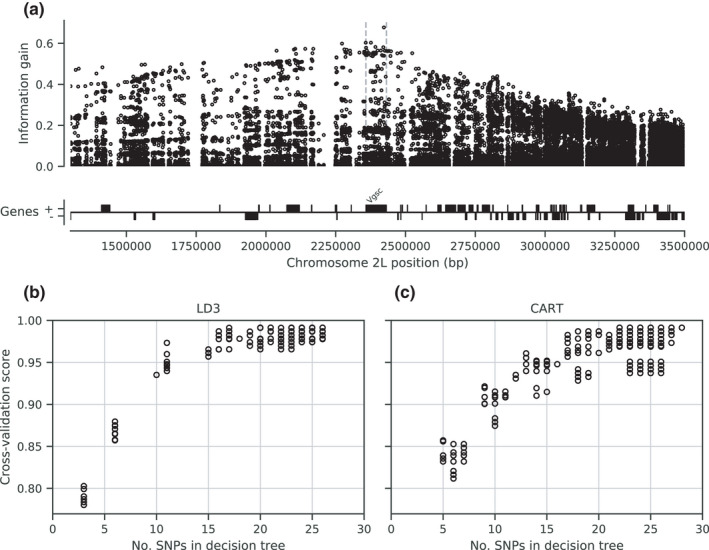
Informative SNPs for haplotype surveillance. a, Each data point represents a single SNP. The information gain value for each SNP provides an indication of how informative the SNP is likely to be if used as part of a genetic assay for testing whether a mosquito carries a resistance haplotype, and if so, which haplotype group it belongs to. b, Number of SNPs required to accurately predict which group a resistance haplotype belongs to. Each data point represents a single decision tree. Decision trees were constructed using either the LD3 (left) or CART (right) algorithm for comparison. Accuracy was evaluated using 10‐fold stratified cross‐validation

To facilitate the development of targeted genetic assays for surveillance of *Vgsc*‐mediated pyrethroid resistance, we have produced several supplementary data tables. In Supplementary Table 1, we list all 82 non‐synonymous variants found within the *Vgsc* gene in this study, with population allele frequencies. In Table S2, we list 756 biallelic SNPs, within the *Vgsc* gene and up to 10 kbp upstream or downstream, that are potentially informative regarding which haplotype group a resistance haplotype belongs to, and thus could be used for tracking the spread of resistance. This table includes the allele frequency within each of the 10 haplotype groups defined here, to aid in identifying SNPs that are highly differentiated between two or more haplotype groups. We also provide Table S3 which lists all 10,244 SNPs found within the *Vgsc* gene and up to 10 kbp upstream or downstream, which might need to be taken into account as flanking variation when searching for PCR primers to amplify a SNP of interest. To provide some indication for how many SNPs would need to be assayed in order to track the spread of resistance, we used haplotype data from this study to construct decision trees that could classify which of the 12 groups a given haplotype belongs to (Figure 6). This analysis suggested that it should be possible to construct a decision tree able to classify haplotypes with >95% accuracy by using 20 SNPs or less. In practice, more SNPs would be needed, to provide some redundancy and also to type non‐synonymous polymorphisms in addition to identifying the genetic background. However, it is still likely to be well within the number of SNPs that could be assayed in a single multiplex via amplicon sequencing. Thus, it should be feasible to produce low‐cost, high‐throughput genetic assays for tracking the spread of pyrethroid resistance. If combined with whole‐genome sequencing of mosquitoes at sentinel sites, this should also allow the identification of newly emerging resistance outbreaks.

## 4 METHODS

### 4.1 Code

All scripts and Jupyter Notebooks used to generate analyses, figures and tables are available from the GitHub repository https://github.com/malariagen/ag1000g‐phase2‐vgsc‐report.

### 4.2 Data

We used variant calls and phased haplotype data from the Ag1000G Phase 2 AR1 data release (https://www.malariagen.net/data/ag1000g‐phase‐2‐ar1). Variant calls from Ag1000G Phase 2 are also available from the European Nucleotide Archive (ENA; http://www.ebi.ac.uk/ena) under study PRJEB36277.

### 4.3 Data collection and processing

For detailed information on Ag1000G WGS sample collection, sequencing, variant calling, quality control and phasing, see (The Anopheles gambiae 1000 Genomes Consortium, 2020; The Anopheles gambiae 1000 Genomes Consortium, 2017). In brief, *An. gambiae* and *An. coluzzii* mosquitoes were collected from 33 sites in 13 countries across Sub‐Saharan Africa: Angola, Bioko, Burkina Faso, Cameroon, Côte d'Ivoire, Gabon, The Gambia, Ghana, Guinea, Guinea‐Bissau, Kenya, Mayotte and Uganda. From Angola and Côte d'Ivoire, just *An. coluzzii* were sampled; Burkina Faso, Ghana and Guinea had samples of both *An. gambiae* and *An. Coluzzii;* and all other populations consisted of purely *An. gambiae*, except for The Gambia, Guinea‐Bissau and Kenya where species status is uncertain (The Anopheles gambiae 1000 Genomes Consortium, 2020). Mosquitoes were individually whole genome sequenced on the Illumina HiSeq 2000 platform, generating 100 bp paired‐end reads. Sequence reads were aligned to the *An. gambiae* AgamP3 reference genome assembly (Holt et al., 2002). Aligned bam files underwent improvement, before variants were called using GATK UnifiedGenotyper. Quality control included removal of samples with mean coverage <= 14x and filtering of variants with attributes that were correlated with Mendelian error in genetic crosses.

The Ag1000G variant data were functionally annotated using the SnpEff v4.1b software (Cingolani et al., 2012). Non‐synonymous *Vgsc* variants were identified as all variants in AgamP4.12 transcript AGAP004707‐RD with a SnpEff annotation of ‘missense’. The *Vgsc* gene is known to exhibit alternative splicing (Davies et al., 2007a); however, at the time of writing the *An. gambiae* gene annotations did not include the alternative transcripts reported by Davies et al. We wrote a Python script to check for the presence of variants that are synonymous according to transcript AGAP004707‐RD but non‐synonymous according to one of the other transcripts present in the gene annotations or in the set reported by Davies et al. Supplementary Table 1 includes the predicted effect for all SNPs that are non‐synonymous in one or more of these transcripts. None of the variants that are non‐synonymous in a transcript other than AGAP004707‐RD were found to be above 5% frequency in any population.

For ease of comparison with previous work on *Vgsc*, pan Insecta, in Table 1 and Table S1, we report codon numbering for both *An. gambiae* and *Musca domestica* (the species in which the gene was first discovered). The *M. domestica Vgsc* sequence (EMBL accession X96668 (Williamson et al., 1996)) was aligned with the *An. gambiae* AGAP004707‐RD sequence (AgamP4.12 geneset) using the Mega v7 software package (Kumar et al., 2016). A map of equivalent codon numbers between the two species for the entire gene can be download from the MalariaGEN website (https://www.malariagen.net/sites/default/files/content/blogs/domestica_gambiae_map.txt).

Haplotypes for each chromosome of each sample were estimated (phased) using phase informative reads (PIRs) and SHAPEIT2 v2.r837 (Delaneau et al., 2013); see (The Anopheles gambiae 1000 Genomes Consortium, 2017) supplementary text for more details. The SHAPEIT2 algorithm is unable to phase multi‐allelic positions; therefore, the two multi‐allelic non‐synonymous SNPs within the *Vgsc* gene, altering codons V402 and M490, were phased onto the biallelic haplotype scaffold using MVNcall v1.0 (Menelaou & Marchini, 2013). Lewontin's *D*′ (Lewontin, 1964) was used to compute the linkage disequilibrium (LD) between all pairs of non‐synonymous *Vgsc* mutations.

### 4.4 Haplotype networks

Haplotype networks were constructed using the median‐joining algorithm (Bandelt et al., 1999) as implemented in a Python module available from https://github.com/malariagen/ag1000g‐phase2‐vgsc‐report. Haplotypes carrying either L995F or L995S mutations were analysed with a maximum edge distance of two SNPs. Networks were rendered with the Graphviz library and a composite figure constructed using Inkscape. Non‐synonymous edges were highlighted using the SnpEff annotations (Cingolani et al., 2012).

### 4.5 Positive selection

Core haplotypes were defined on a 6,078‐bp region spanning *Vgsc* codon 995, from chromosome arm 2L position 2,420,443 and ending at position 2,426,521. This region was chosen as it was the smallest region sufficient to differentiate between the ten genetic backgrounds carrying either of the known resistance alleles L995F or L995S. Extended haplotype homozygosity (EHH) was computed for all core haplotypes as described in Sabeti et al. (2002) using scikit‐allel version 1.1.9 (Miles & Harding, 2016), excluding non‐synonymous and singleton SNPs. Analyses of haplotype homozygosity in moving windows (Figures S1 and S2) and pairwise haplotype sharing (Figure S3) were performed using custom Python code available from https://github.com/malariagen/ag1000g‐phase2‐vgsc‐report.

### 4.6 Design of genetic assays for surveillance of pyrethroid resistance

To explore the feasibility of identifying a small subset of SNPs that would be sufficient to identify each of the genetic backgrounds carrying known or putative resistance alleles, we started with an input data set of all SNPs within the *Vgsc* gene or in the flanking regions 20 kbp upstream and downstream of the gene. Each of the 2284 haplotypes in the Ag1000G Phase 2 cohort was labelled according to which core haplotype it carried, combining all core haplotypes not carrying known or putative resistance alleles together as a single ‘wild‐type’ group. Decision tree classifiers were then constructed using scikit‐learn version 0.19.0 (Pedregosa et al., 2011) for a range of maximum depths, repeating the tree construction process 10 times for each maximum depth with a different initial random state. The classification accuracy of each tree was evaluated using stratified fivefold cross‐validation.

### 4.7 Homology modelling

A homology model of the *An. gambiae* voltage‐gated sodium channel (AGAP004707‐RD AgamP4.12) was generated using the 3.8 Å resolution structure of the *Periplaneta americana* sodium channel NavPaS structure (PDB code 5X0M) (Shen et al., 2017). Sequences were aligned using Clustal Omega (Sievers et al., 2011). 50 starting models were generated using MODELLER (Webb et al., 2016). The internal scoring function of MODELLER was used to select 10 models, which were visually inspected and submitted to the VADAR webserver (Willard et al., 2003) to assess stereochemistry in order to select the best final model. Figures were produced using PyMOL (DeLano Scientific, San Carlos, CA, USA).

## AUTHOR CONTRIBUTIONS

C.S.C., A.M., M.J.D., D.W. and D.K. designed the study. C.S.C., A.M., N.J.H. and A.O.O. carried out the analysis. The Ag1000G Consortium undertook collection, preparation, sequencing and primary analysis of the samples. C.S.C., A.M. and M.J.D. wrote the manuscript. All authors read and approved the final manuscript.

## Supporting information

Fig S1‐S3

Table S1

Table S3

Table S2

## Data Availability

Sequence read alignments and variant calls from Ag1000G phase 2 are available from the European Nucleotide Archive under study accession PRJEB36277 (ENA ‐ http://www.ebi.ac.uk/ena). Sequence read alignments for samples in Ag1000G phase 1 are available under study accession PRJEB18691.

## Footnotes

^1^
Codon numbering is given here relative to transcript AGAP004707‐RD as defined in the AgamP4.12 gene‐set annotations. A mapping of codon numbers from AGAP004707‐RD to *Musca domestica*, the system in which *kdr* mutations were first described (Williamson et al., 1996), is given in Table 1.
