# Supplementary material for: The genetic architecture of target‐site resistance to pyrethroid insecticides in the African malaria vectors Anopheles gambiae and Anopheles coluzzii
Source: Mol Ecol. 2021 Mar 8;30(21):5303–17. doi: 10.1111/mec.15845 (PMC9019111; doi:10.1111/mec.15845)
Supplement: Supplementary file 1 — Fig S1‐S3 [file MEC-30-5303-s002.pdf]

Supplementary Figures

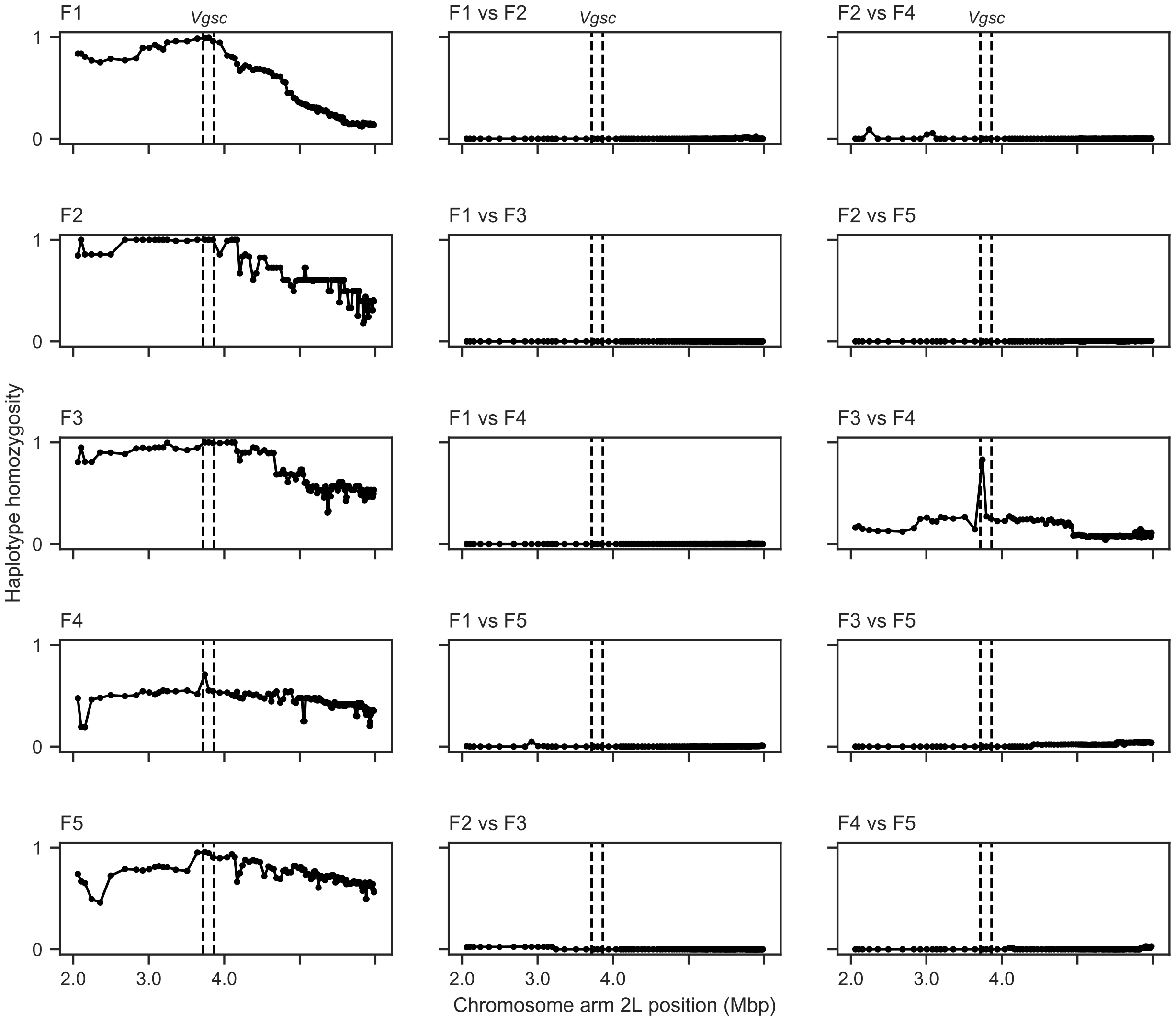

Figure 1. Windowed analysis of haplotype homozygosity for genetic backgrounds carrying the `L995F` allele. Each sub-plot shows the fraction of haplotype pairs that are identical within half-overlapping moving windows of 1000 SNPs. Each sub-plot in the left-hand column shows homozygosity for haplotype pairs within one of the haplotype groups identified by the network analysis. Sub-plots in the central and right-hand columns show homozygosity for haplotype pairs between two haplotype groups. If two haplotype groups are truly unrelated, haplotype homozygosity between them should be close to zero across the whole genome region. Dashed vertical lines show the location of the *Vgsc* gene.

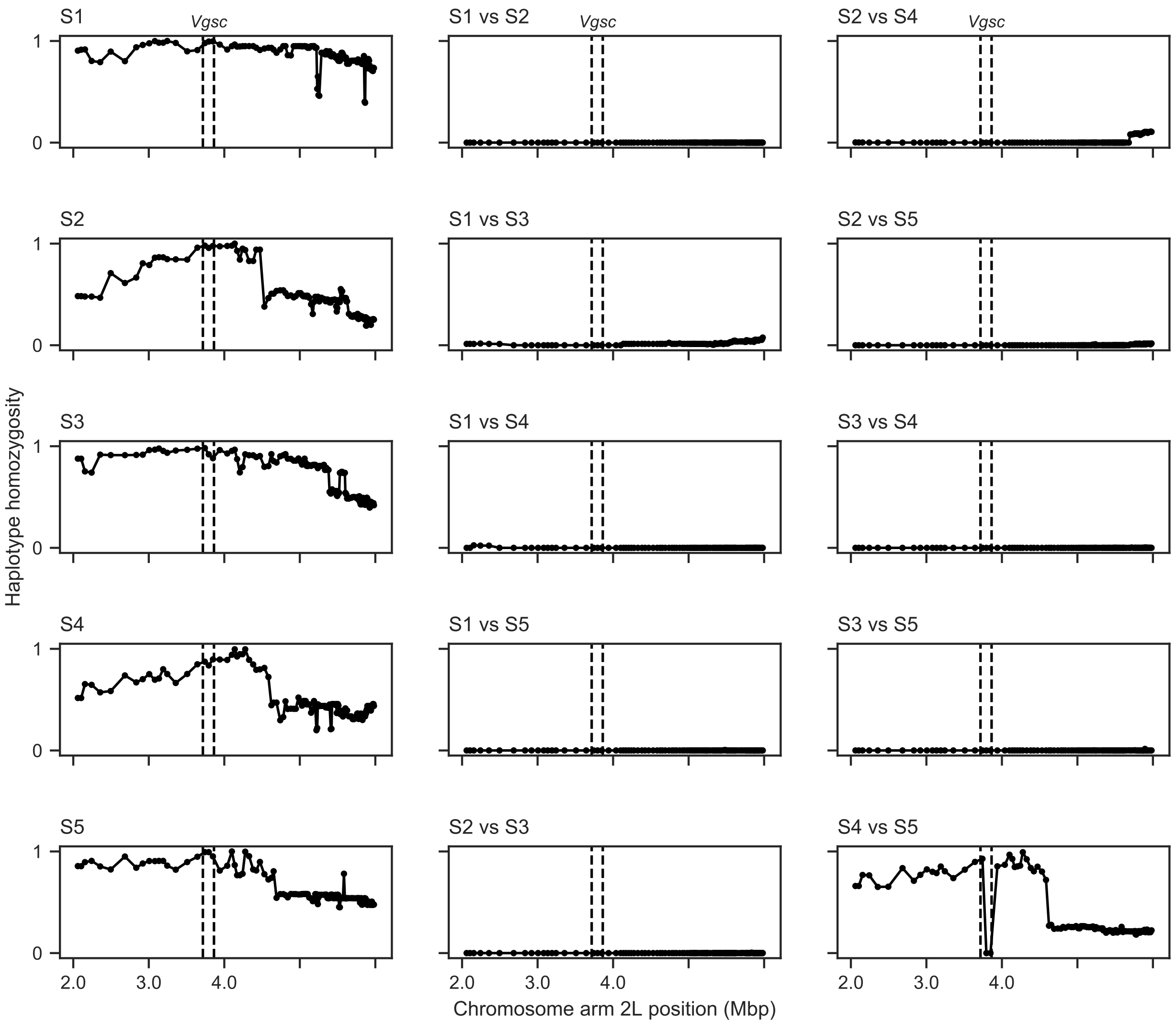

Figure 2. Windowed analysis of haplotype homozygosity for genetic backgrounds carrying the L995S allele. See Supplementary Figure 1 for explanation. Haplotype homozygosity is high between groups S4 and S5 on both flanks of the gene, indicating that haplotypes from both groups are in fact closely related.

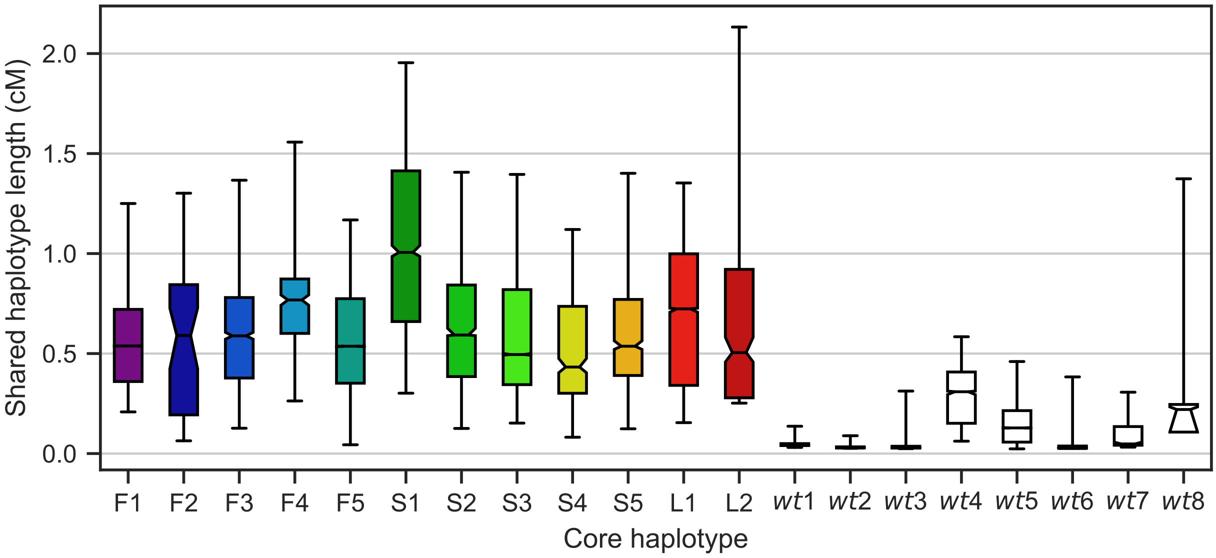

Figure 3. Shared haplotype length. Each bar shows the distribution of shared haplotype lengths between all pairs of haplotypes with the same core haplotype. For each pair of haplotypes, the shared haplotype length is computed as the region extending upstream and downstream from the core locus (*Vgsc* codon 995) over which haplotypes are identical at all non-singleton variants. The *Vgsc* gene sits on the border of pericentromeric heterochromatin and euchromatin, and we assume different recombination rates in upstream and downstream regions. The shared haplotype length is expressed in centiMorgans (cM) assuming a constant recombination rate of 2.0 cM/Mb on the downstream (euchromatin) flank and 0.6 cM/Mb on the upstream (heterochromatin) flank. Bars show the inter-quartile range, fliers show the 5-95th percentiles, horizontal black line shows the median, notch in bar shows the 95% bootstrap confidence interval for the median. Haplotypes F1-5 each carry the `L995F` resistance allele. Haplotypes S1-5 each carry the `L995S` resistance allele. Haplotype L1 carries the `I1527T` allele. Haplotype L2 carries the `M490I` allele. Wild-type (*wt*) haplotypes do not carry any known or putative resistance alleles.
